# The hr1 and Fusion Peptide Regions of the Subgroup B Avian Sarcoma and Leukosis Virus Envelope Glycoprotein Influence Low pH-Dependent Membrane Fusion

**DOI:** 10.1371/journal.pone.0000171

**Published:** 2007-01-24

**Authors:** Angeline Rose Babel, James Bruce, John A.T. Young

**Affiliations:** 1 McArdle Laboratory for Cancer Research, Department of Oncology, University of Wisconsin-Madison, Madison, Wisconsin, United States of America; 2 Institute for Molecular Virology, Bock Laboratories, University of Wisconsin-Madison, Madison, Wisconsin, United States of America; 3 Infectious Disease Laboratory, The Salk Institute for Biological Studies, La Jolla, California, United States of America; AIDS Research Center, China

## Abstract

The avian sarcoma and leukosis virus (ASLV) envelope glycoprotein (Env) is activated to trigger fusion by a two-step mechanism involving receptor-priming and low pH fusion activation. In order to identify regions of ASLV Env that can regulate this process, a genetic selection method was used to identify subgroup B (ASLV-B) virus-infected cells resistant to low pH-triggered fusion when incubated with cells expressing the cognate TVB receptor. The subgroup B viral Env (*envB*) genes were then isolated from these cells and characterized by DNA sequencing. This led to identification of two frequent EnvB alterations which allowed TVB receptor-binding but altered the pH-threshold of membrane fusion activation: a 13 amino acid deletion in the host range 1 (hr1) region of the surface (SU) EnvB subunit, and the A32V amino acid change within the fusion peptide of the transmembrane (TM) EnvB subunit. These data indicate that these two regions of EnvB can influence the pH threshold of fusion activation.

## Introduction

Avian Sarcoma and Leukosis Virus (ASLV)-receptor interactions are a useful model system for studying the mechanism of retroviral entry into cells since there are multiple virus subgroups (designated A–J) that use different cellular receptors [1]: TVA for ASLV-A [2], TVB for ASLV-B, ASLV-D, and ASLV-E [3]–[5], TVC for ASLV-C [6], and the chicken Na^+^/H^+^ exchanger type 1 for ASLV-J [7].

ASLV entry is mediated by the metastable viral envelope glycoprotein (Env), comprising a surface subunit (SU), which binds receptor, and a transmembrane subunit (TM). Receptor-interacting determinants have been previously mapped to three variable regions (designated as hr1, hr2, and vr3) of ASLV SU [8]–[16]. The TM protein anchors Env in the viral membrane and contains an internal fusion peptide located inside its amino terminus [17]–[20]. TM also contains two heptad regions, designated as HR1 and HR2, which come together forming a six-helix bundle or hairpin during membrane fusion [21], [22]. The current model of ASLV entry invokes structural changes in SU which are induced upon receptor-binding (receptor-priming) leading to TM adopting a pre-hairpin conformation with its fusion peptide inserted in the target membrane [17], [23]–[25]. Evidence either for or against lipid-mixing at this stage has been presented [22], [26]–[28]. Low pH is required to drive six-helix bundle formation leading to the completion of membrane fusion [22], [24], [27]–[29].

In an effort to explore the ASLV Env fusion mechanism in more detail we set out to identify determinants of Env, which influence low pH-dependent fusion. By using a genetic approach that employed a cell-cell fusion assay we have identified mutations in the hr1 subregion of SU, and within the fusion peptide of TM, which render Env less sensitive to fusion activation at low pH, implicating these two regions in regulating ASLV Env-driven membrane fusion.

## Methods

### Cell lines and Viruses

Chicken DF-1 cells and 293:TVB^S3^ *Δ*DD cells were described previously [5], [30]. The subgroup B ASLV vector, RCASH-B, encoding hygromycin B phosphotransferase was described previously [31]. DF-1 cells were transfected with the RCASH-B vector using the calcium phosphate method and cells chronically infected with the virus were selected in medium containing 300 µg/ml hygromycin B.

### Genetic Selection and Flow Cytometry

Approximately 1×10^6^ DF-1 cells that were chronically infected with RCASH-B, and selected in medium containing 300 µg/ml hygromycin B, were plated with 9×10^6^ 293:TVB^S3^ *Δ*DD cells. The cells were incubated together for 4 hours at 37^o^C, treated with 20 mM MES buffer (pH 5.6) at 37°C to induce cell-cell fusion for 90 mins and then placed under selection in medium containing 300 µg/ml hygromycin B. This procedure was repeated six times giving rise to the R6 (pH 5.6) cell population. Flow cytometry to determine EnvB surface expression was performed with a TVB-immunoadhesin (TVB^S3^-IgG) and with a FITC-conjugated secondary antibody (Dako, Denmark) as described previously [5].

### Quantification of R6 (pH 5.6) cell resistance to syncytia formation

R6 (pH 5.6) cells were plated with 293:TVB^S3^ *Δ*DD cells at ratios varying from 1∶10 to 1∶10^6^ with the total cell number in each population held constant at 2×10^6^ cells per well. After 4 hours at 37°C, the cells were treated with medium buffered with 20 mM MES pH 5.6 for 90 min at 37°C and then incubated in medium containing 300 µg/ml hygromycin B for 14 days. Hygromycin B-resistant colonies were stained with 1% methylene blue/20% 2-propanol/5% acetic acid. Wells containing distinct colonies were counted and the numbers obtained were corrected using the following formula: N×(2×10^6^/R) where N = number of colonies, and R = number of R6 (pH 5.6) cells plated).

### PCR amplification and DNA sequencing

Single cell clones that were resistant to syncytia formation were isolated from the R6 (pH 5.6) population. The *envB* genes contained in these cells were isolated by PCR amplification from cellular genomic DNA [5′-acggtaccgatcaagcatggcatttctgactggataccctgg-3′ (sense primer, KpnI site underlined) and 5′-acactagtgatgccacagtggtacgcgagg-3′ (antisense primer, SpeI site underlined)] and were subcloned into KpnI/SpeI digested pCI plasmid (Invitrogen, LaJolla, CA). The DNA sequences of the *envB* genes were determined using Big Dye sequencing (Applied Biosystems, Foster City, CA). PCR amplification was also used to screen individual single cell clones for the *Δ*152–164 mutation using primers (5′-cagaactacaactgctagg-3′) and (5′-cggtttcgaggagttagagg-3′) which generates either a 209 bp (wild-type) or a 170 bp (*Δ*152–164 mutant) product.

### Wild-type and mutant EnvB protein function

Wild-type and mutant *envB* genes were inserted upstream of the internal ribosome entry site (ires)- enhanced green fluorescent protein (eGFP) cassette in the murine leukemia virus-based retroviral vector pCMMP.IRES.eGFP [32]The MLV vectors encoding the different envB proteins were produced in the extracellular supernatants of transiently transfected 293 cells as described previously [33] and used to infect DF-1 cells. The eGFP positive cells were sorted 48 hours later on a FACSDiva (University of Wisconsin Comprehensive Cancer Center, Madison, WI). Cell surface expression and receptor-binding of each altered EnvB protein was confirmed by flow cytometric analysis as before. These cells expressing altered EnvB proteins were mixed at a 1∶10 ratio with 293 TVB^S3^ *Δ*DD cells that had been labeled with 40 ng/ml rhodamine 18 (R18) for 30 minutes (Invitrogen, LaJolla, CA). After 4 hours, medium buffered with MES at pH 5.6, 5.0, or 4.6, was added for 10 minutes at 37°C and cell-cell fusion was monitored by fluorescence microscopy using an Axiovert25 fluorescent microscope 4–6 hours later.

## Results

**Figure 1:**
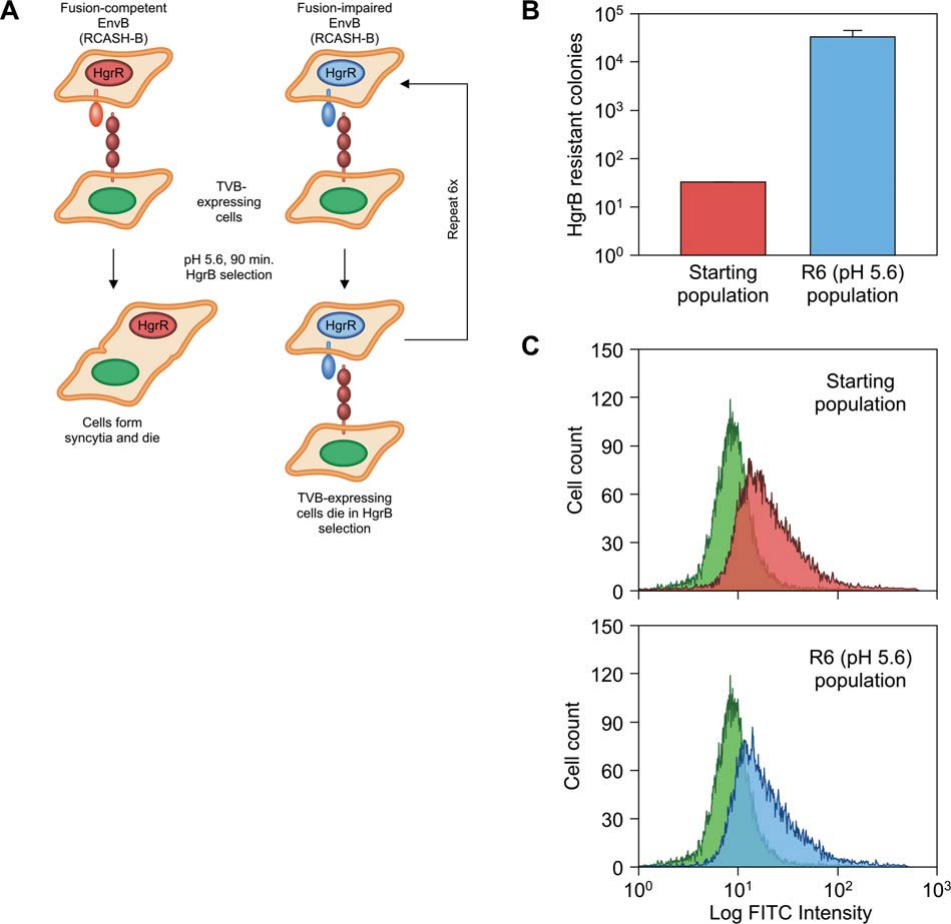
Selection of ASLV-B infected DF-1 cells that do not undergo low pH-mediated cell-cell fusion. (A) Selection scheme used to identify subgroup B ASLV-infected cells that are resistant to low pH-mediated syncytia formation. (B) The numbers of hygromycin B-resistant virus-infected colonies that resulted from cell-cell fusion experiments preformed with 293:TVB^S3^ *Δ*DD cells and either starting population of RCASH-B infected cells or R6 (pH 5.6) cells, are shown. This experiment was performed in triplicate and the average mean values obtained are shown along with the standard deviation of the data. (C) Flow cytometric analysis of EnvB expression. Uninfected cells (green histogram), the starting population of RCASH-B infected DF-1 cells (red histogram, upper panel) and the R6 (pH 5.6) cells (blue histogram, lower panel) were incubated with TVB^S3^-rIgG and a FITC-conjugated anti-rabbit antibody and analyzed by flow cytometry as described previously [5], [27].

In order to identify determinants of ASLV Env that influence receptor-priming and low pH fusion activation, a genetic approach was used. The principle of this approach, which relied on the error-prone nature of reverse transcription (error rate between 10^−4^ and 10^−5^) [34] to generate mutations during virus replication and is outlined in Fig. 1A. Briefly, we hypothesized that within a starting population of cells that are chronically infected with a replication-competent subgroup B ASLV vector (encoding hygromycin B phosphotransferase) [31], there would be a subpopulation of cells containing viral variants harboring mutations which render Env unable to support low pH-dependent membrane fusion. If so, we reasoned that it should be possible to selectively amplify this class of cells by incubating the whole virus-infected cell population with an excess of uninfected cells that express the cognate TVB receptor, inducing cell-cell fusion at low pH, and then incubating the cells with medium containing hygromycin B. Under these conditions virus-infected cells resistant to cell-cell fusion would be selectively amplified: cells that underwent fusion would give rise to a non-viable syncytium and excess TVB-expressing cells that had not undergone fusion would be eliminated from the culture by the hygromycin B selection.

To test this idea, chicken DF-1 cells that were chronically infected with the subgroup B ASLV vector, RCASH-B, encoding hygromycin B phosphotransferase [31] were incubated briefly at pH 5.6 with a 9-fold excess of 293:TVB^S3^ *Δ*DD cells that express a cytoplasmic tail-truncated form of the TVB receptor [3]. The mixed cell population was then placed under selection in medium containing 300 µg/ml hygromycin B. This procedure was repeated six times giving rise to a population of infected DF-1 cells that were highly resistant to low pH-induced syncytia formation (R6 (pH 5.6) cell population). Flow cytometric analysis performed with a TVB-immunoadhesin (TVB^S3^-IgG) [35] and with a FITC-conjugated secondary antibody confirmed that cells of the R6 (pH 5.6) cells expressed, on their surfaces, forms of EnvB that were competent for binding to the TVB receptor (Fig. 1B).

To determine their level of resistance to low pH-induced fusion, R6 (pH 5.6) cells were incubated briefly at pH 5.6 with different ratios of 293:TVB^S3^ *Δ*DD cells before selecting in medium containing 300 µg/ml hygromycin B. The resultant hygromycin B-resistant colonies serve as a measure of the number of non-fused virus-infected cells. Based upon this analysis the R6 (pH 5.6) cells were estimated to be approximately 1000-fold more resistant to low pH induced cell-cell fusion when compared to the starting population of virus-infected cells (Fig. 1C).

**Figure 2:**
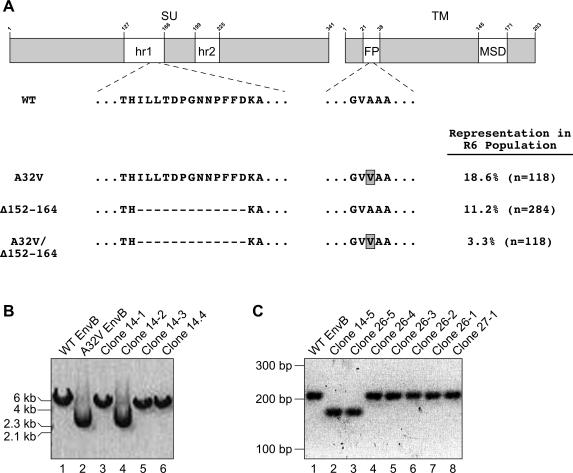
Common *envB* mutations in the R6 (pH 5.6) cell population. (A) Schematic of the EnvB protein showing host range regions hr1and hr2 of SU and the fusion peptide (FP) and membrane spanning domain (MSD) of TM. The amino acid sequences of wild-type (WT) Env B and of three common mutations found in the R6 (pH 5.6) cell population are shown below. The frequencies of each mutation in the R6 (pH 5.6) population are indicated in parentheses, measured as described in the text (n = number of cloned copies of the *envB* genes that were characterized). (B) Representative screen for the A32V mutation. Individual plasmid DNAs containing *envB* genes were screened for the A32V mutation by digestion with PvuII as described in the text. Plasmids containing wild-type *envB* gave rise to a linear 6 kb DNA fragment (lanes 1, 3, 5, and 6) whereas those containing A32V *envB* remain undigested (lanes 2 and 4). Lanes 1 and 2: wild-type and A32V *envB* controls. Lanes 4–6: Individual *envB* genes cloned from the R6 (pH 5.6) population. (C) Representative PCR-amplification based screen for the *Δ*152–164 mutation as described in the text. Plasmid DNA containing wild-type *envB* give rise to a 209 bp DNA fragment (lanes 1 and 4–8), while those containing the *Δ*152–164 mutant give rise to a 170 bp DNA fragment (lanes 2 and 3). Lane 1; wild-type *envB* control, Lanes 2–7: Individual *envB* genes cloned from the R6 (pH 5.6) population.

To identify mutations in the *envB* gene that are responsible for the altered fusion phenotype, single cell clones that were resistant to syncytia formation were isolated from the R6(pH 5.6) population. The full-length *envB* genes in these cells were isolated by PCR amplification and their DNA sequences were determined. A total of 22 distinct mutations were identified, most of which were represented once amongst the clones that were analyzed (data not shown). However, three mutations were found in multiple clones and were chosen for further study. One mutation was a single nucleotide change (A to T) at position 1312 in the *envB* gene that led to the loss of a PvuII site and replacement of alanine 32 in the TM subunit with a valine (A32V) (Fig. 2A). The second mutation was a deletion of nucleotides 632 to 671 of *envB* resulting in a 13 amino acid deletion (residues 152–164) within the hr1 region of SU (Fig. 2A) (*Δ*152–164). The third mutant *envB* gene contained both the A32V and *Δ*152–164 mutations (Fig. 2A). These mutants were chosen for additional analysis because they were found in more than one independently-isolated Env DNA fragment and thus they could not have resulted from an error during PCR amplification.

To assess the frequency of these mutations in the selected cell population, *envB* genes were isolated by PCR amplification from the bulk R6 (pH 5.6) cell population and subcloned into the pCI vector, and individual bacterial transformants were screened for the A32V mutation by PvuII digestion: plasmid DNA containing wild-type *envB* is cut once (linear 6 kb DNA) whereas the mutant A32V *envB* gene is resistant to digestion (uncut/supercoiled) (Fig. 2B). Transformants were also screened for the *Δ*152–164 mutation by a PCR amplification method that generated either a 209 bp (wild-type) or a 170 bp (*Δ*152–164 mutant) product (Fig. 2C). The A32V and *Δ*152–164 mutations were estimated to be present in the R6 (pH 5.6) cell population at frequencies of 18.6%, and 11.2%, respectively (Fig. 2A) while 3.3% contained both mutations (Fig. 2A).

**Figure 3:**
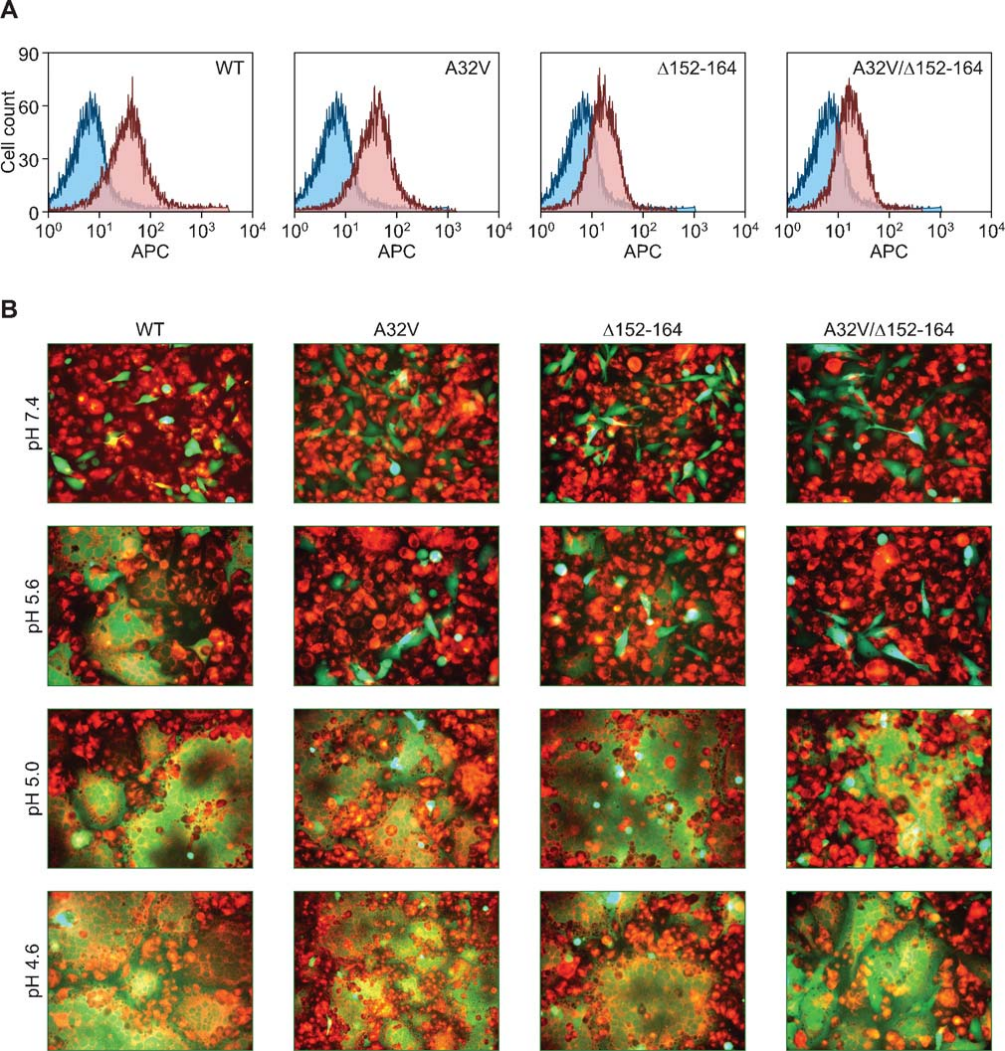
The mutant EnvB proteins exhibit altered low pH thresholds for fusion activation. (A) DF-1 cells (blue histogram) and DF-1 cells expressing wild-type (WT), and either of the mutant forms of EnvB (red histograms) were analyzed by flow cytometry as described in the Fig. 1 legend. (B)The virus-infected DF-1 cells (GFP-positive green) were mixed with 293:TVB^S3^ *Δ*DD cells labeled by expression of eGFP. The 293:TVB^S3^ *Δ*DD cells were labeled with R18 red. Images were taken using an Axiovert25 microscope at 50× magnification. Shown are representative panels from the whole cell cultures.

To test the function of the altered Env proteins, mutant *envB* genes were inserted upstream of the internal ribosome entry site (ires)- enhanced green fluorescent protein (eGFP) cassette in the murine leukemia virus-based retroviral vector pCMMP.IRES.eGFP [32]. These constructs were then used to transduce DF-1 cells that were then sorted for GFP expression by FACS. Cell surface expression and the receptor-binding ability of each altered EnvB protein was then confirmed by flow cytometric analysis using TVB^S3^-IgG and a FITC-conjugated secondary antibody. (Fig. 3A)

These cells were then incubated under different low pH conditions at a 1∶10 ratio with rhodamine-labeled 293 TVB^S3^ *Δ*DD cells. Cell-cell fusion was then monitored by fluorescence microscopy. This analysis revealed that the altered EnvB proteins are resistant to fusion activation at pH 5.6 as expected (Fig. 3B). However, each EnvB protein was competent to elicit cell-cell fusion when the cells were incubated at pH 5.0 or below (Fig. 3B). These studies confirm that these EnvB mutations alter the pH threshold of EnvB fusion activation by making this process dependent upon more acidic conditions.

## Discussion

In this report we took advantage a genetic screen that used cell-cell fusion as the basis for selection to identify mutations in two regions of ASLV-B Env that have a significant impact on the pH threshold of membrane fusion activation. The first of these is the deletion of a 13 amino acid segment (residues 152–164) from the hr1 region of SU. The second is the A32V amino acid substitution within the internal fusion peptide region of TM. Either of these mutations, or both in combination, changes the pH threshold of ASLV Env so that fusion is driven only under more acidic conditions than are required for the wild-type viral glycoprotein. Since type I viral glycoproteins are converted from a metastable native state to a much more stable state after fusion activation [36], we postulate that the increased acid requirement exhibited by the mutant EnvB proteins may be due to their increased stability relative to the wild-type glycoprotein.

Previously, the hr1 region of ASLV SU has been shown to harbor determinants that dictate receptor binding and usage [8]–[10], [12]–[14], [16]. In this study we have identified a 13 amino acid deletion within this region of EnvB, which still permits TVB receptor-binding but renders Env more resistant to fusion activation at low pH. This apparent change in Env stability might indicate hr1 determinant involvement in fusogenic activation of the viral glycoprotein. Indeed, based upon the expanded host cell-tropism associated with an hr1 mutation, L154S, it has been similarly proposed that this region of EnvB might be involved in fusogenic activation [37]. Altered Env stability might also account for the TVA-independent infection seen with viruses bearing a six amino acid deletion within the hr1 region of ASLV-A SU [12].

The finding that the A32V amino acid change in the fusion peptide of the EnvB TM protein alters the pH threshold of fusion activation is similar to that made with influenza A virus hemagglutinin HA2 subunit [38], [39]. Since the A32 residue is conserved in the Env proteins of other ASLV subgroups [18], it may play a conserved role in ASLV fusion. Consistently, we have found that cells expressing A32V subgroup A ASLV Env are incapable of mediating cell-cell fusion with TVA-expressing cells at low pH even though the Env protein is expressed on the cell surface and is capable of binding to soluble TVA receptor (data not shown). Future studies will be aimed at determining how the hr1 and fusion peptide regions contribute to the pH threshold of ASLV Env activation.

## References

[1] Barnard RJ, Elleder D, Young JA (2006). Avian sarcoma and leukosis virus-receptor interactions: from classical genetics to novel insights into virus-cell membrane fusion.. Virology.

[2] Bates P, Young JA, Varmus HE (1993). A receptor for subgroup A Rous sarcoma virus is related to the low density lipoprotein receptor.. Cell.

[3] Brojatsch J, Naughton J, Adkins HB, Young JA (2000). TVB receptors for cytopathic and noncytopathic subgroups of avian leukosis viruses are functional death receptors.. J Virol.

[4] Adkins HB, Brojatsch J, Naughton J, Rolls MM, Pesola JM (1997). Identification of a cellular receptor for subgroup E avian leukosis virus.. Proc Natl Acad Sci U S A.

[5] Adkins HB, Brojatsch J, Young JA (2000). Identification and characterization of a shared TNFR-related receptor for subgroup B, D, and E avian leukosis viruses reveal cysteine residues required specifically for subgroup E viral entry.. J Virol.

[6] Elleder D, Stepanets V, Melder DC, Senigl F, Geryk J (2005). The receptor for the subgroup C avian sarcoma and leukosis viruses, Tvc, is related to mammalian butyrophilins, members of the immunoglobulin superfamily.. J Virol.

[7] Chai N, Bates P (2006). Na^+^/H^+^ exchanger type 1 is a receptor for pathogenic subgroup J avian leukosis virus.. Proc Natl Acad Sci U S A.

[8] Dorner AJ, Coffin JM (1986). Determinants for receptor interaction and cell killing on the avian retrovirus glycoprotein gp85.. Cell.

[9] Bova CA, Manfredi JP, Swanstrom R (1986). env genes of avian retroviruses: nucleotide sequence and molecular recombinants define host range determinants.. Virology.

[10] Bova CA, Olsen JC, Swanstrom R (1988). The avian retrovirus env gene family: molecular analysis of host range and antigenic variants.. J Virol.

[11] Damico R, Rong L, Bates P (1999). Substitutions in the receptor-binding domain of the avian sarcoma and leukosis virus envelope uncouple receptor-triggered structural rearrangements in the surface and transmembrane subunits.. J Virol.

[12] Holmen SL, Federspiel MJ (2000). Selection of a subgroup A avian leukosis virus [ALV(A)] envelope resistant to soluble ALV(A) surface glycoprotein.. Virology.

[13] Holmen SL, Melder DC, Federspiel MJ (2001). Identification of key residues in subgroup A avian leukosis virus envelope determining receptor binding affinity and infectivity of cells expressing chicken or quail Tva receptor.. J Virol.

[14] Melder DC, Pankratz VS, Federspiel MJ (2003). Evolutionary pressure of a receptor competitor selects different subgroup a avian leukosis virus escape variants with altered receptor interactions.. J Virol.

[15] Rong L, Edinger A, Bates P (1997). Role of basic residues in the subgroup-determining region of the subgroup A avian sarcoma and leukosis virus envelope in receptor binding and infection.. J Virol.

[16] Taplitz RA, Coffin JM (1997). Selection of an avian retrovirus mutant with extended receptor usage.. J Virol.

[17] Hernandez LD, White JM (1998). Mutational analysis of the candidate internal fusion peptide of the avian leukosis and sarcoma virus subgroup A envelope glycoprotein.. J Virol.

[18] Balliet JW, Gendron K, Bates P (2000). Mutational analysis of the subgroup A avian sarcoma and leukosis virus putative fusion peptide domain.. J Virol.

[19] Delos SE, Gilbert JM, White JM (2000). The central proline of an internal viral fusion peptide serves two important roles.. J Virol.

[20] Delos SE, White JM (2000). Critical role for the cysteines flanking the internal fusion peptide of avian sarcoma/leukosis virus envelope glycoprotein.. J Virol.

[21] Netter RC, Amberg SM, Balliet JW, Biscone MJ, Vermeulen A (2004). Heptad repeat 2-based peptides inhibit avian sarcoma and leukosis virus subgroup a infection and identify a fusion intermediate.. J Virol.

[22] Markosyan RM, Bates P, Cohen FS, Melikyan GB (2004). A study of low pH-induced refolding of Env of avian sarcoma and leukosis virus into a six-helix bundle.. Biophys J.

[23] Damico RL, Crane J, Bates P (1998). Receptor-triggered membrane association of a model retroviral glycoprotein.. Proc Natl Acad Sci U S A.

[24] Mothes W, Boerger AL, Narayan S, Cunningham JM, Young JA (2000). Retroviral entry mediated by receptor priming and low pH triggering of an envelope glycoprotein.. Cell.

[25] Delos SE, Godby JA, White JM (2005). Receptor-induced conformational changes in the SU subunit of the avian sarcoma/leukosis virus A envelope protein: implications for fusion activation.. J Virol.

[26] Earp LJ, Delos SE, Netter RC, Bates P, White JM (2003). The avian retrovirus avian sarcoma/leukosis virus subtype A reaches the lipid mixing stage of fusion at neutral pH.. J Virol.

[27] Melikyan GB, Barnard RJ, Markosyan RM, Young JA, Cohen FS (2004). Low pH is required for avian sarcoma and leukosis virus Env-induced hemifusion and fusion pore formation but not for pore growth.. J Virol.

[28] Melikyan GB, Barnard RJ, Abrahamyan LG, Mothes W, Young JA (2005). Imaging individual retroviral fusion events: from hemifusion to pore formation and growth.. Proc Natl Acad Sci U S A.

[29] Matsuyama S, Delos SE, White JM (2004). Sequential roles of receptor binding and low pH in forming prehairpin and hairpin conformations of a retroviral envelope glycoprotein.. J Virol.

[30] Himly M, Foster DN, Bottoli I, Iacovoni JS, Vogt PK (1998). The DF-1 chicken fibroblast cell line: transformation induced by diverse oncogenes and cell death resulting from infection by avian leukosis viruses.. Virology.

[31] Young JA, Bates P, Varmus HE (1993). Isolation of a chicken gene that confers susceptibility to infection by subgroup A avian leukosis and sarcoma viruses.. J Virol.

[32] Bruce JW, Bradley KA, Ahlquist P, Young JA (2005). Isolation of cell lines that show novel, murine leukemia virus-specific blocks to early steps of retroviral replication.. J Virol.

[33] Boerger AL, Snitkovsky S, Young JA (1999). Retroviral vectors preloaded with a viral receptor-ligand bridge protein are targeted to specific cell types.. Proc Natl Acad Sci U S A.

[34] Katz RA, Skalka AM (1990). Generation of diversity in retroviruses.. Annu Rev Genet.

[35] Adkins HB, Blacklow SC, Young JA (2001). Two functionally distinct forms of a retroviral receptor explain the nonreciprocal receptor interference among subgroups B, D, and E avian leukosis viruses.. J Virol.

[36] Earp LJ, Delos SE, Park HE, White JM (2005). The many mechanisms of viral membrane fusion proteins.. Curr Top Microbiol Immunol.

[37] Rainey GJ, Natonson A, Maxfield LF, Coffin JM (2003). Mechanisms of avian retroviral host range extension.. J Virol.

[38] Steinhauer DA, Wharton SA, Skehel JJ, Wiley DC (1995). Studies of the membrane fusion activities of fusion peptide mutants of influenza virus hemagglutinin.. J Virol.

[39] Daniels RS, Downie JC, Hay AJ, Knossow M, Skehel JJ (1985). Fusion mutants of the influenza virus hemagglutinin glycoprotein.. Cell.

